# An inpatient antimicrobial stewardship team driven penicillin allergy delabeling protocol for minimal and low-risk penicillin allergic patients

**DOI:** 10.1017/ash.2025.7

**Published:** 2025-02-17

**Authors:** Shivanjali Shankaran, Emily Adochio, Robert Petrak, Benjamin Goldenberg, Fischer Herald, Christy Lunn, Hayley Hodgson, Sarah Won

**Affiliations:** 1 Division of Infectious Diseases, Rush University Medical Center, Chicago, IL, USA; 2 Department of Pharmacy, Rush University Medical Center, Chicago, IL, USA

## Abstract

Inappropriate penicillin allergy labeling results in suboptimal or excessive broad spectrum antibiotic use. In this multidisciplinary project, the antimicrobial stewardship team safely delabeled 71.4% of hospitalized patients approached. Similar programs may also be able to delabel minimal or low-risk penicillin allergic patients without formal allergy consultation.

## Introduction

Penicillin (PCN) allergy prevalence has been overestimated. While 10% of the population report an allergy, <1% have a true allergy.^1^ A PCN allergy label leads to increased use of broad-spectrum antibiotics, drug resistance, hospital stays, and all-cause mortality.^2–4^ It is therefore imperative to delabel those that do not have a PCN allergy. Prior studies^5^ have demonstrated the safety of PCN or amoxicillin oral challenges in patients with low-risk allergies, without a need for PCN skin testing. Of those receiving oral amoxicillin challenges, <5% have delayed reactions. These oral challenges are also cheaper and simpler than PCN skin testing, with significant cost savings.^6^ Due to ease of one-time oral amoxicillin challenges, delabeling opportunities can be expanded to ID clinicians and pharmacists on antimicrobial stewardship (ASP) teams. With their expertise, they are well poised to further bolster delabeling efforts, with previous studies showing successful delabeling by ID^7^ as well as ASP. Here we describe our experience with delabeling adult inpatients with minimal or low-risk PCN allergies at a 664-bed academic facility in Chicago, IL.

## Methods

**Figure 1.**
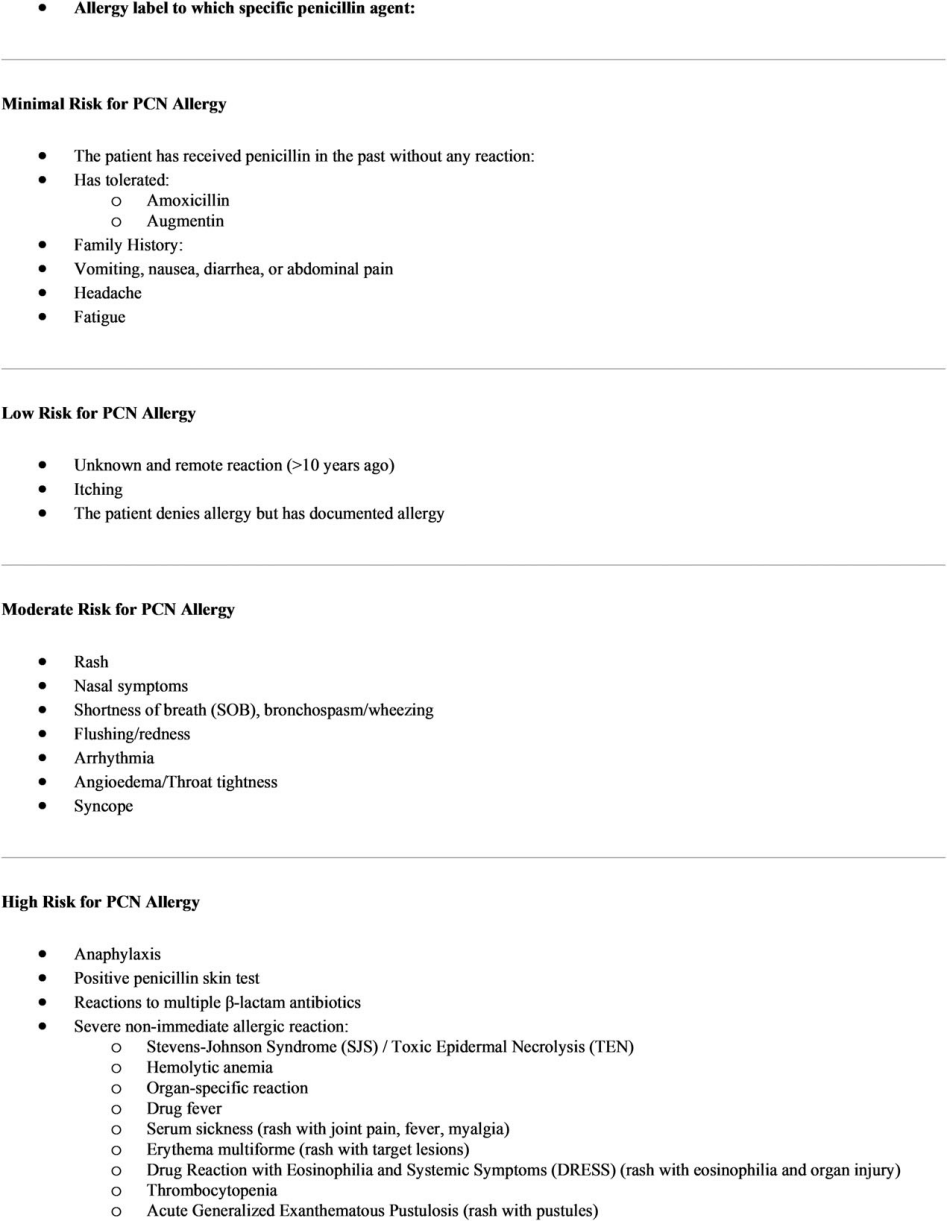
Penicillin allergy questionnaire ASP - PCN allergy note.

**Figure 2.**
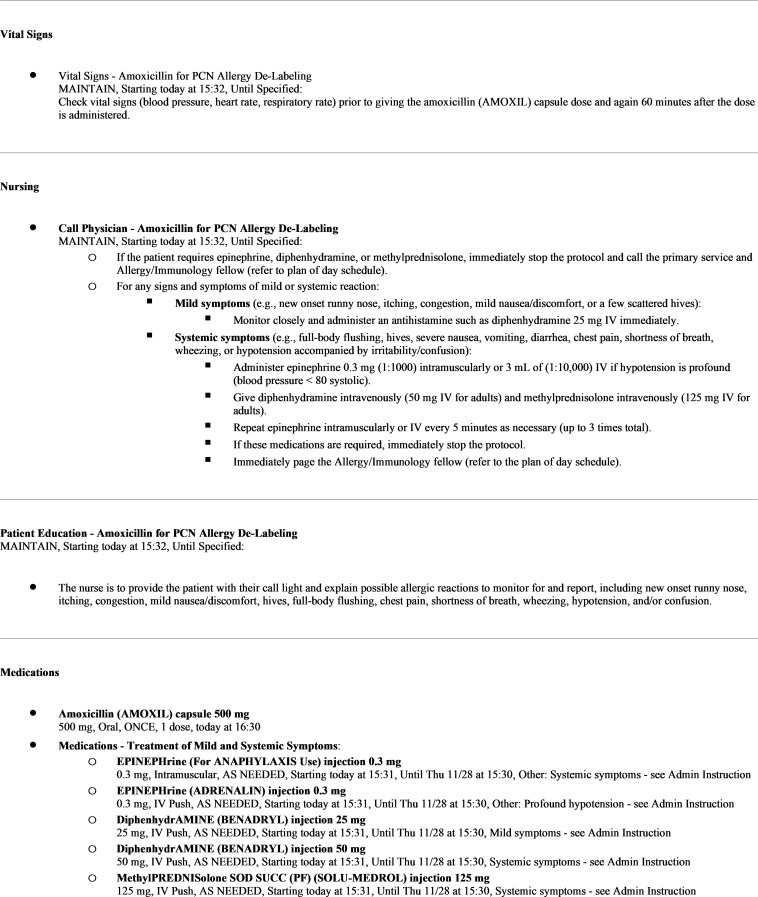
Amoxicillin challenge order set restrictions for use: Infectious disease or allergy and immunology amoxicillin for PCN allergy de-labeling module.

This quality improvement project was initiated by ID trained physician leaders of the ASP team to bring antibiotic prescribing in PCN allergic patients in concordance with updated allergy management recommendations.^8^ A questionnaire defining minimal, low, and moderate risk PCN allergy was devised using a toolkit from this same publication, in collaboration with the Department of Allergy and Immunology (Figure 1). After approval was obtained from the Antimicrobial Stewardship Subcommittee and Pharmacy, Nutrition and Therapeutics Committee, multidisciplinary educational sessions were held with nursing, hospitalists, and internal medicine residents to ensure buy-in and comfort with the process. A pre-existing amoxicillin graded challenge order set was modified for the “Amoxicillin for PCN Delabeling” module (Figure 2). This included a 500 mg oral amoxicillin order, vital sign monitoring parameters and orders for rescue medications for nurses in case of an allergic reaction.

Between June 2022 and February 2023, the ASP team (ASP pharmacists, stewardship physicians, ID fellows, pharmacy residents, and medicine residents) used electronic medical records (EMR) to prospectively identify hospitalized patients >18 years of age with documented PCN allergy on adult internal medicine floors at Rush University Medical Center. After chart review, patients who seemed to have an unclear or minimal or low-risk PCN allergy were approached by the ASP team during normal working hours. Based on verbal history and previous antibiotic records, patients were stratified into risk categories using the above questionnaire. Minimal risk patients were delabeled either by history alone or by documented prescription of amoxicillin without allergic reaction, or if requested, after an amoxicillin challenge. Low-risk patients were offered an amoxicillin challenge. All other patients were excluded.

In April 2023, all delabeled patients’ charts were reviewed to assess for PCN allergy relabeling at subsequent hospitalizations.

## Interventions

### Amoxicillin challenge

Low-risk patients, or minimal risk patients by request, underwent an amoxicillin challenge. ASP collaborated with the patient’s inpatient healthcare team who placed the “Amoxicillin for PCN delabeling” order within the EMR. Nurses then administered amoxicillin and monitored vital signs at the time of administration and then again 60 minutes later

The ASP team conducted a chart assessment to monitor for adverse effects within 24 hours after the amoxicillin challenge.

### Penicillin delabeling

Patients who exhibited no adverse events during or within 24 hours of amoxicillin challenge were revisited by the ASP team. They were then delabeled following discussion and mutual agreement with the patient. For all delabeled patients, the ASP team updated the EMR allergy documentation, deleting the allergy entry, and incorporating the dates of delabeling with a rationale. A note about the delabeling was added to the patient’s discharge instructions and to the patient’s EMR. The inpatient team and the patient’s primary care physician were informed of the delabeling.

## Results

Fifty two patient charts were reviewed. Of them, 42 patients were appropriate for delabeling, with 21 being minimal risk and 21 being low risk. For patients where demographic data was available, 59.5% were female (22 of 37), 61.7% were Black (21 of 34), and 11.76% were Latine (4 of 34). Thirty of 42 (71.4%) were successfully delabeled; the remaining 12 were not delabeled, either due to patient discomfort or hospital discharge prior to intervention. Of 30 delabeled patients, 14 were delabeled by history alone; all had tolerated oral amoxicillin before. Of the remaining sixteen, 12 low-risk patients and 4 minimal risk patients underwent amoxicillin challenge. Amoxicillin challenges were well tolerated, and all 16 were successfully delabeled. Only one patient was relabeled at a subsequent hospitalization.

## Discussion

In our pilot project, we were able to safely and easily delabel 71.4% of approached patients, demonstrating that PCN delabeling programs can be successfully deployed in hospitalized settings. Forty seven percent were delabeled by history alone, with the remainder delabeled by a onetime order placed by primary teams and administered by bedside nurses. Additionally, of 30 delabeled patients, only one was subsequently relabeled. We believe this durability was due to our multidisciplinary approach involving the patient, primary team, and EMR documentation. As this project demonstrates the importance and simplicity of history taking in PCN allergy delabeling programs, it can be adapted to use locally available resources. It can be comfortably expanded to hospitalists and advanced practice providers on the inpatient setting, with the goal of delabeling minimal or low-risk PCN allergies without the need for specialized resources.
